# Supplementary material for: Identification of photoperiod-induced specific miRNAs in the adrenal glands of Sunite sheep (Ovis aries)
Source: Front Vet Sci. 2022 Jul 22;9:888207. doi: 10.3389/fvets.2022.888207 (PMC9354845; doi:10.3389/fvets.2022.888207)
Supplement: Supplementary file 1 [file Data_Sheet_1.ZIP › Supplementary file/miRDeep2 file.zip!/miRDeep2 file/Know_Structure/SP42A2/oar-mir-10a.pdf]

miRBase precursor : oar-mir-10a
Total read count : 135221
oar-miR-10a read count : 134275
remaining reads : 946

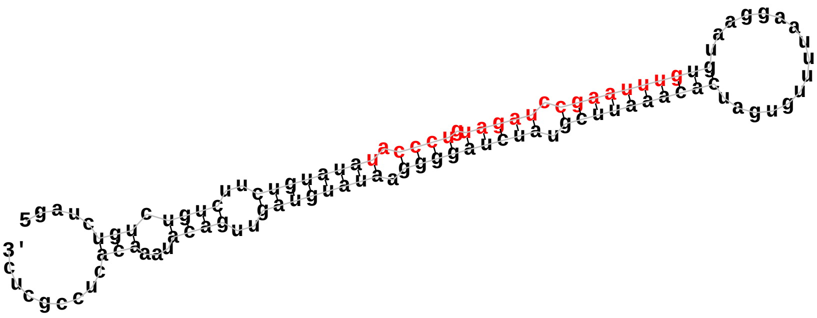

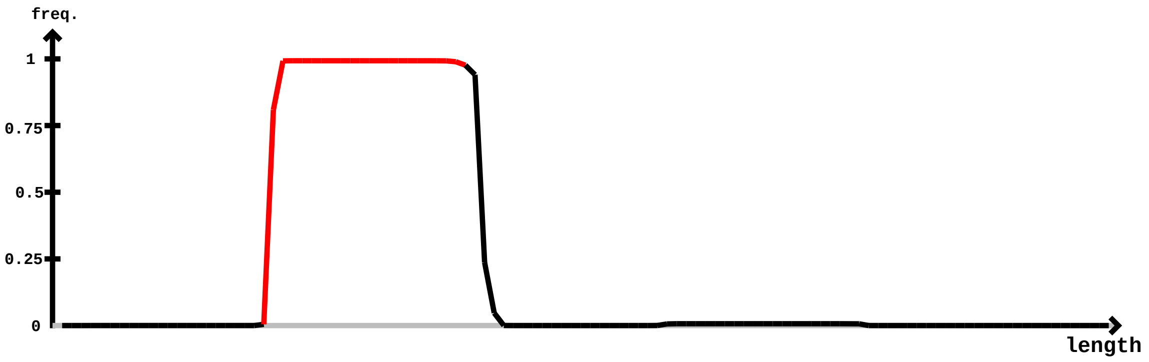

oar-miR-10a

5'- gaucugucugucuucuguauauacccuguagauccgaauuuguguaaggaauuuugugaucacaaauucguaucuaggggaauauguaguugacauaaacacuccgcuc -3'	exp		
....(((.((((..((((((((.((((.(((((.((((((((((...............)))))))))).))))))))).))))))))..))))...)))..........	reads	mm	sample
.aucugucugucuucuguaua..	4	0	seq
..ucugucuUucuucuguaua..	1	1	seq
..ucugucugucuucuguaua..	3	0	seq
...cugucugucuucuguaua..	1	0	seq
...................uauacccuguagauccgaauuug...	1	0	seq
....................auacccuguagauccgaau..	2	0	seq
....................auacccuguagauccgaauu...	2	0	seq
....................auacccuguagauccgaauuu..	40	0	seq
....................auacccuguagauccgaauuug...	80	0	seq
....................auacccuguagauccgaauuugu..	458	0	seq
....................auacccuguagauccgaauuugug...	25	0	seq
....................auacccuguagauccgaauuugugu..	5	0	seq
.....................uacccuguagauccgaa...	10	0	seq
.....................uacccuguagauccgaau..	47	0	seq
.....................uacccuguagauccgaauu...	363	0	seq
.....................uacccuguagauccgaauuu..	1509	0	seq
.....................uacccuguagauccgaauuug...	4094	0	seq
.....................uacccuguagauccgaauuugu..	82236	0	seq
.....................uacccuguagauccgaauuugug...	17052	0	seq
.....................uacccuguagauccgaauuugugu..	3423	0	seq
.....................uacccuguagauccgaauuugugua...	27	0	seq
.....................uacccuguagauccgaauuuguguaa..	2	0	seq
.....................uacccuguagauccgaauuuguguaagg..	1	0	seq
......................acccuguagauccgaau..	4	0	seq
......................acccuguagUuccgaauu...	1	1	seq
......................acccuguagauccgaauu...	67	0	seq
......................acccuguagauccgaauuu..	219	0	seq
......................acccuguagauccgaauuug...	666	0	seq
......................acccuguagauccgaauuugu..	12458	0	seq
......................acccuguagauccgaauuugug...	8509	0	seq
......................acccuguagauccgaauuugugu..	2895	0	seq
......................acccuguagauccgaauuugugua...	13	0	seq
.......................cccuguagauccgaauuugu..	19	0	seq
.......................cccuguagauccgaauuugug...	31	0	seq
.......................cccuguagauccgaauuugugu..	10	0	seq

oar-miR-10a

```
gaucugucugucuucuguauauacccuguagauccgaauuuguguaaggaauuuugugaucacaaauucguaucuaggggaauauguaguugacauaaacacuccgcuc
.......................cccuguagauccgaauuugugua...............................................................    1    0    seq
........................ccuguagauccgaauuugu..................................................................    3    0    seq
........................ccuguagauccgaauuugug.................................................................    1    0    seq
........................ccuguagauccgaauuugugu................................................................    1    0    seq
.........................cuguagauccgaauuugu..................................................................    1    0    seq
........................................................ugaucacaaauucguaucuaggggaau..........................    1    0    seq
...........................................................ucacaaauucguaucuagggga............................    2    0    seq
...........................................................ucacaaauucguaucuaggggaa...........................    1    0    seq
.............................................................acaaauucguaucuaggggaa...........................    1    0    seq
.............................................................acaaauucguaucuaggggaau..........................    27    0    seq
.............................................................acaaauucguaucuaggggaaua.........................    2    0    seq
..............................................................caaauucguaucuagggg.............................    1    0    seq
..............................................................caaauucguaucuagggga............................    23    0    seq
..............................................................caaauucguaucuaggggaa...........................    31    0    seq
..............................................................caaauucguaucuaggggaau..........................    730    0    seq
..............................................................caaauucguaucuaggggaaua.........................    8    0    seq
..............................................................caaauucguaucuaggggaauau........................    1    0    seq
...............................................................aaauucguaucuaggggaa...........................    2    0    seq
...............................................................aaauucguaucuaggggaau..........................    98    0    seq
...............................................................aaauucguaucuaggggaaua.........................    1    0    seq
...............................................................aaauucguaucuaggggaauau........................    3    0    seq
................................................................aauucguaucuaggggaau..........................    2    0    seq
................................................................aauucguaucuaggggaauau........................    1    0    seq
..................................................................uucguaucuaggggaaua.........................    1    0    seq
```
